# Annotations

**Published:** 1902-07-05

**Authors:** 


### Annotations.

### Medical Politics at the Antipodes.

In the Colonies many experiments in sociology are possible which cannot be easily conducted to a final issue in old countries. Hence both the medical profession and the managers of the great friendly societies in England will naturally watch with considerable interest the contest which is at present raging in New South Wales between the local branch of the British Medical Association and a great friendly society called the Australian Natives' Association; especially since the point at issue is exactly that which is the perennial *casus belli* between similar associations in this country, namely, the wage limit. The level at which it is proposed to draw the line is different from what it is here; the proposal of the Medical Association being to exclude from medical benefits all those whose incomes exceed £200 per annum, which is a good deal higher than would be regarded as satisfactory in England. This is doubtless due to differences in social customs and the value of money, but the principle is the same, the Natives' Association saying, as do our friendly societies, that they can draw no line of distinction between their more and their less successful members. As things stand at present the British Medical Association is pretty strong in the colony, and it seems to be having things pretty much its own way, but there is talk of the Natives' Association forming a medical board of their own, and importing medical men from England, and if this should be done it will be interesting to see whether it is possible for a compact body like a Colonial branch of the British Medical Association to rule the roost.

### Alcoholism and its Cures.

Among the many ways of producing in the drunkard a dislike for alcohol, one of the simplest is to "physic" his drink. The trick is as old as the hills, and so long as the nauseating effect of his "doctored" potations are believed by the drinker to be the inevitable and unavoidable result of alcohol, he will probably avoid indulgence in alcoholic stimulants. If at the same time one could instil into the drunkard's mind a deep and undoubting conviction that any reversion to drinking habits will be attended by the direst consequences, this belief also, so long as it lasted, would clearly be a direct incentive to prolonged abstinence. Just as signing the pledge will keep many a man from that "first glass" which in so many cases leads straight to a wild orgie, so may the belief that alcohol is dangerous to him, act as an effectual check, and preserve him from starting again on his downward path. Moreover, we can quite understand that if, while this little hocus pocus is being performed upon him, he is made to take some medicine or undergo some wonderful process by which he is assured that he will be cured, he will be still further strengthened in his determination, for faith in physic seems ineradicable from the human mind. Hence, although no one can doubt that certain "cures" for inebriety of which one hears a good deal are based upon deceit, we do not want to throw doubt upon the statement that in certain cases they have helped alcoholics to throw off their evil habits, at any rate for a time. When, however, we are told that these cures which depend upon the use of certain secret remedies have a power for good which is not possessed by those used by orthodox physicians, it is time to inquire how these vaunted remedies are administered, and if, as we are assured is the case in some instances, while the medicines given are being represented as of wonderful efficacy, the alcohol taken by the patient is being doctored in such a way as to create nausea and a consequent disgust for alcohol, then it must be broadly stated that the whole affair is a piece of charlatanism. That such performances sometimes succeed has nothing to do with the matter. So long as a man refrains from alcohol, whether he is led to do so by hope of heaven or by fear of stomach ache, he at least is cured of his inebriety. But that is a very different thing from curing alcoholism by the administration of a drug.

### South African Typhoid.

The War Office is doubtless an admirable mill, but it grinds with an appalling slowness, and we think that Dr. Leigh Canney has justice and reason on his side in pointing out that the loss of life from typhoid fever in South Africa, which has been even greater this year than it was last, has been largely due to the delay of the War Office in considering the scheme laid before it by him for providing all troops on the march with a supply of safe water. Some time in July last Dr. Canney put forward his scheme, and although in August it was declared by Lord Stanley to be "entirely impracticable for active service," the suggestion was kept before the authorities. In November it was discussed at the Royal United Service Institution, and in the same month Dr. Canney received a letter from the Commander-in-Chief stating that it was "receiving careful consideration and will be again brought before the Army Medical Advisory Board." At last, in May, the House of Commons was informed that measures were being taken to carry out a scheme which had been prepared. Meanwhile the epidemic went on. Dr. Canney says that the mobile columns have suffered from 20 to 30 deaths daily for months during the past winter, which has been equivalent to some 6,000 to 10,000 men constantly ill in hospital, most of which might have been avoided if his scheme had been accepted at once. Truly the War Office grinds wondrous slow. For what was Dr. Canney's invention? Was it some new and untried speculation? Nothing of the sort. What Dr. Canney urged was that we should earnestly and carefully, and as part of our military system, apply and put in operation for the benefit of our troops a certain little bit of elementary and universally accepted sanitary knowledge, nothing more abstruse than this, that one cannot catch typhoid from freshly-boiled water. Dr. Canney holds that, with the lives of so many troops in the balance, so soon as he propounded his plan a commission ought to have been appointed to investigate it, and should have sat eight hours a day until they had finished the work. The very mention of such a suggestion is enough to make the War Office shiver. What did happen was that the commission which had to consider the matter "did not meet until late in November, and then only once a fortnight, and the scheme is not yet in working order."

